# Compound heterozygosity for hemoglobin S and hemoglobin E in a family of Proto-Australoid origin: a case report

**DOI:** 10.1186/s13256-021-02974-4

**Published:** 2021-08-02

**Authors:** Noymi Basumatary, Dipankar Baruah, Paresh Kumar Sarma, Jatin Sarmah

**Affiliations:** 1 Department of Biotechnology, Bodoland University, Kokrajhar, Assam India; 2 Department of Pathology, Fakhruddin Ali Ahmed Medical College and Hospital, Barpeta, Assam India; 3 Department of Medicine, Fakhruddin Ali Ahmed Medical College and Hospital, Barpeta, Assam India; 4 Department of Biotechnology and Co-ordinator, DBT (Govt. of India) sponsored Bioinformatics Infrastructure Facility, Bodoland University, Kokrajhar, Assam India

**Keywords:** Proto-Australoid, Hemoglobinopathy, Hemolytic anemia, Sickle cell disease, Tea tribe

## Abstract

**Background:**

Hemoglobin S and E are commonly occurring hemoglobin variants among distinctly separate tribal populations of Central and Northeast India, respectively. Combined heterozygosity for hemoglobin S and E or hemoglobin SE disease is a benign clinical condition with rare incidence. Reports of approximately 46 hemoglobin SE cases are available worldwide. We conducted a screening program to study the prevalence of hemoglobin variants among the tribal population working in the tea estates of Northeast India. A total of 551 subjects were screened, and complete blood count was performed. Based on their hematological profiles, hemoglobin typing was done for 218 subjects.

**Case presentation:**

We describe a case of an adolescent male of Munda tribe diagnosed as double heterozygous for hemoglobin S and E. On screening of the nuclear family of the subject, the mother was found to have hemoglobin E disease and father as hemoglobin S trait. Both siblings of the subject were diagnosed as hemoglobin E trait.

**Conclusion:**

This is the first case of compound heterozygous for hemoglobin S and E to be reported from the tea tribes of Assam, India.

## Background

Hemoglobinopathies are monogenic disorders characterized by abnormal hemoglobin structure [1]. Among the hemoglobin variants, the most commonly occurring and clinically significant variants are hemoglobin S (Hb S), hemoglobin C (Hb C), hemoglobin E (Hb E), and thalassemia [2]. In context to its occurrence, Hb E is the second most common abnormal variant of hemoglobin in the world and most common variant in Southeast Asia [3]. Central-West Africa, East Asia, and India experience higher occurrence of sickle cell disease in comparison with other parts of the world. Hemoglobinopathies are a cause of both economic and psychosocial burden [4]. Sickle cell disease shows an autosomal recessive inheritance resulting from A > T mutation in the sixth residue of the β-globin chain. Hb E results from a Glu→Lys mutation in the 26th amino acid.

Among the different types of hemoglobinopathies, prevalence of Hb S and Hb E in India is 4.3% and 10.9% respectively [5]. The burden of hemoglobinopathies in India is so high that it has become a major public health issue in some parts of the country [3]. In India, prevalence of Hb S among the tribals of central, southern, and western part has been reported [6]. In the eastern and northeastern part, Hb E is prevalent [7]. Sickle cell disease, particularly, has turned into a major health concern in states such as Chhattisgarh, Maharashtra, Gujarat, Jharkhand, Madhya Pradesh, and Odisha, demanding serious attention.

A large workforce is employed in the tea estates of Assam, which constitutively forms the tea-tribe community. These laborers were brought to Assam by British rulers from the present states of Chhattisgarh, West Bengal, Odisha, and Jharkhand during the last part of the nineteenth century. Ethnically, they belong to the Proto-Australoid race and are of Austro-Asiatic language group. High prevalence of sickle cell anemia among the Adivasi (ab-origin) population of mainland India has been reported [6]. We conducted a study to investigate the prevalence of sickle cell disorder among the tea estate laborers of Udalguri District of Assam, India.

## Case presentation

**Fig. 1:**
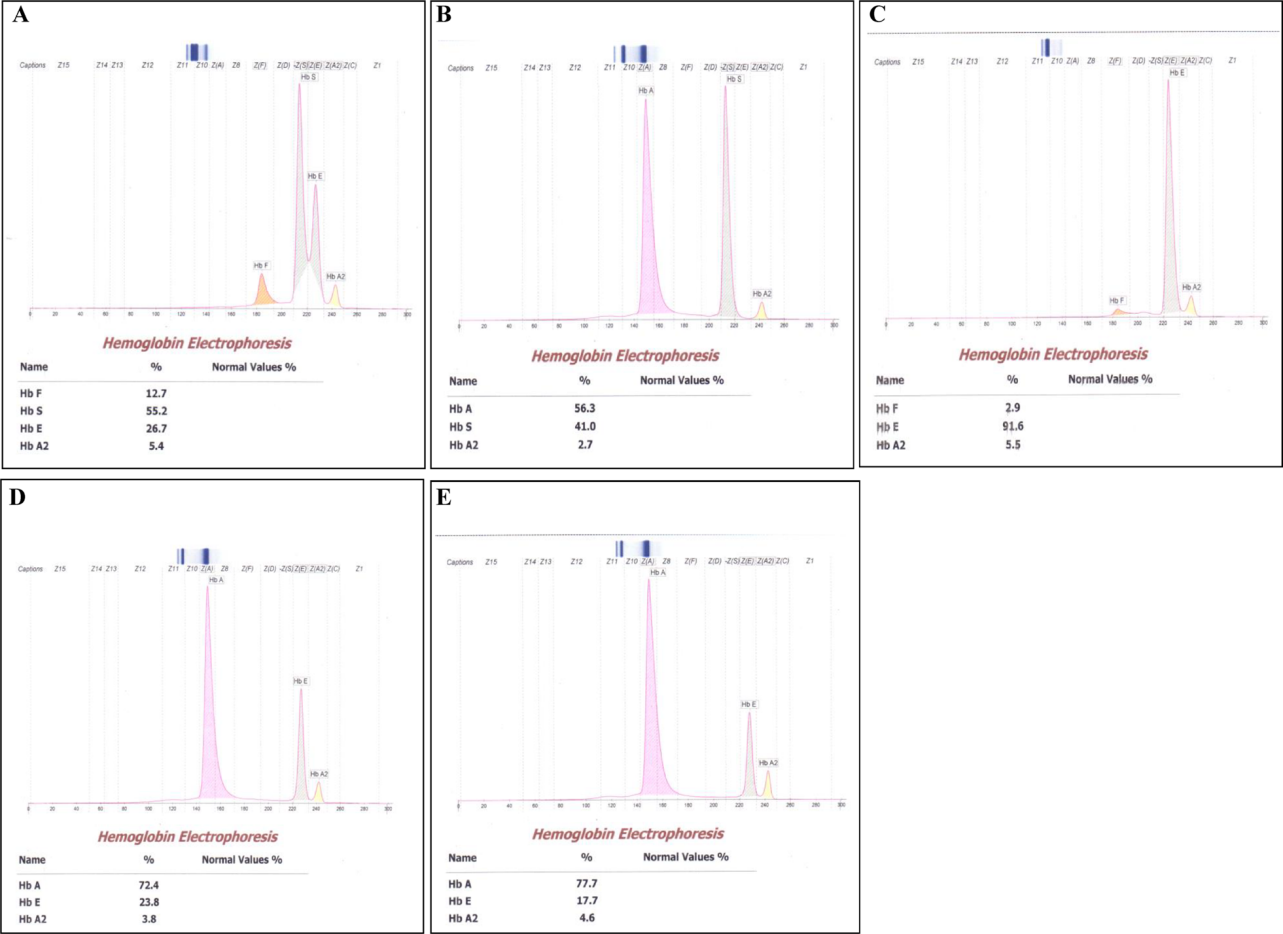
Hb-electrophoresis chromatogram of family members of the Hb SE case. **A** Hb SE subject, **B** Hb S heterozygous father, **C** Hb E homozygous mother, **D** Hb E heterozygous sister, **E** Hb E heterozygous brother

The present study was approved by the Institutional Ethics Committee of Bodoland University, Kokrajhar, Assam, India vide Ref. No.:-IEC/BU/ICMR/2019-2 dated 10/05/2019. Consent was obtained from the participants prior to blood collection. Complete blood count (CBC) was conducted for 551 apparently healthy laborers of both sexes. Based on the hematological profiles, namely, hemoglobin (Hb)%, mean corpuscular volume (MCV), mean corpuscular hemoglobin (MCH), and mean corpuscular hemoglobin concentration (MCHC) of the subjects by Sysmex Hematology Analyzer (XP-100), Hb electrophoresis was performed in 218 cases in Sebia Minicap Automated Capillary Electrophoresis system. In an adolescent male of 17 years of age from a Munda family, one peak was detected in the Hb S zone and another in the Hb E zone (Fig. 1A). On the basis of Hb electrophoresis chromatogram, the case was confirmed to be double heterozygous for Hb S and Hb E (Hb SE) trait. Based on the finding, we performed screening for all members of the nuclear family.

**Fig. 2:**
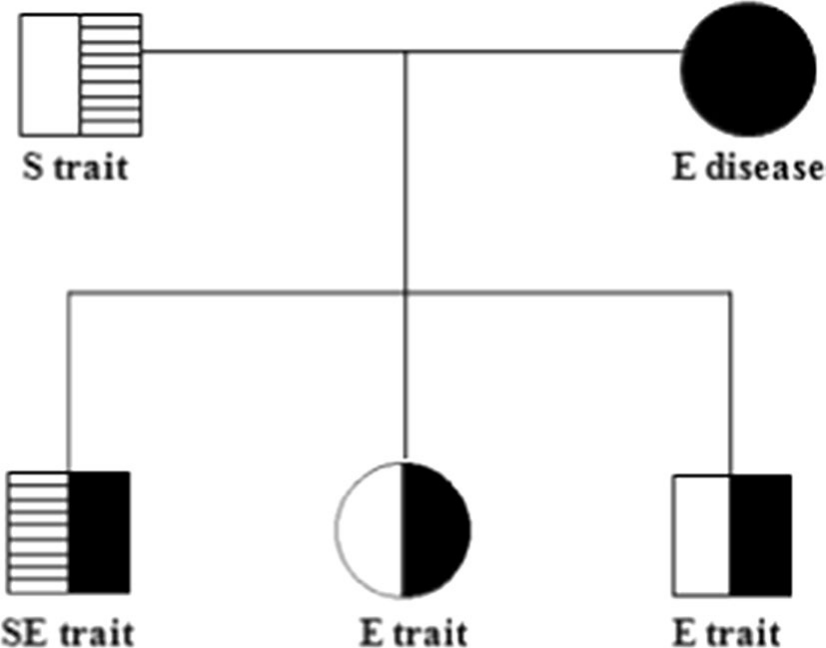
Family pedigree of the Hb SE case

Hematological profiles of the members of the family are presented in Table 1. The case subject showed low Hb (7.8 g/dl), MCHC (29.3%), and RBC count (2.34 million/mm^3^). Other parameters such as MCV (114 fl), MCH (33.3 pg), and red cell distribution width (RDW) (18.5%) were higher than the normal values. Hb electrophoresis showed 12.7% Hb F, 5.4% Hb A_2_, 55.2% Hb S, and 26.7% Hb E. The case subject was an academically average senior secondary student with no previous record of medication. His body weight and height were 63.9 kg and 5′ 7″, respectively. Family screening revealed his father as Hb S heterozygous (Fig. 1B) and mother as Hb E homozygous (Fig. 1C). Both siblings of the subject were also diagnosed as Hb E trait (Fig. 1D, E). Family tree of the Hb SE case is shown in Fig. 2. Collection of marriage history data from the District Marriage Registry Office did not show any record of intermarriage of the case subject’s family with Mongoloid race.

**Table 1:** Hematological profile of the nuclear family of Hb SE case

Parameters	Relation				
	Subject	Father	Mother	Sister	Brother
Hb (g/dl)	7.8	13.9	10.5	7.1	11.6
RBC (million/mm^3^)	2.34	4.92	3.64	3.1	4.34
MCV (fl)	114	94	98	84	93
MCH (pg)	33.3	28.3	28.8	22.7	26.6
MCHC (g/dl)	29.3	30.1	29.4	27	28.6
RDW (%)	18.5	12.1	17.6	22.2	17.4
Hb F (%)	12.7	0.0	2.9	0.0	0.0
Hb A (%)	0.0	56.3	0.0	72.4	77.7
Hb A_2_ (%)	5.4	2.7	5.5	3.8	4.6
Hb S (%)	55.2	41.0	0.0	0.0	0.0
Hb E (%)	26.7	0.0	91.6	23.8	17.7

Hb: hemoglobin; RBC: red blood cell; MCV: mean corpuscular volume; MCH: mean corpuscular hemoglobin; MCHC: mean corpuscular hemoglobin concentration; RDW: red cell distribution width

## Discussion

The tribals of Central India (including Munda tribe) are racially different from the tribals of northeastern part of India. Tribals of Chhattisgarh, West Bengal, Odisha, and Jharkhand are Austro-Asiatic speakers of Proto-Australoid race, whereas the tribals of Northeast India are Tibeto-Burman speaking group of Mongoloid origin. Reports on existence of Hb S and Hb E separately among the tea tribe of Assam are well known [8]. However, compound heterozygosity for Hb S and Hb E was not reported earlier from any community from the multiethnic and multiracial population of Northeast India. Hb SE is a hemoglobin variant having an effect on β-globin gene [9]. Patients detected with Hb SE disease showed a substitution of glutamic acid by lysine at 26th position of β-globin chain [10]. Such double heterozygous condition was reported from a 15-year-old *Teli* male (Other Backward Caste) from Central India who complained of frequent upper respiratory tract infections, weakness, and fatigue [11]. In our report, compound heterozygosity for Hb S and Hb E trait has been presented. Even though the case is anemic, he did not show any other symptoms including transfusion dependency. Unlike other hemoglobinopathies, Hb SE symptoms appear in the late childhood stage or after 20 years [12]. Hematological profile of the subject showed decreased Hb, MCHC, and RBC count and increased MCV, MCH, and RDW values. Hb electrophoresis showed 12.7% of Hb F, 5.4% of Hb A_2_, 55.2% of Hb S, and 26.7% of Hb E. Earlier workers have commented that the hematological parameters and Hb levels do not help in identifying the clinical severity of the condition [13]. Earlier workers also commented that the presence of greater amount of Hb F and lesser amount of Hb S decreases the appearance of clinical symptoms of the patient [14].

Interaction of Hb S heterozygous father with Hb E homozygous mother has given rise to composite heterozygosity for Hb S and Hb E. On investigation of family history for the last five generations, both parents of the subject had no record of intermarriage with the tribals of Mongoloid origin. The case subject’s siblings diagnosed as Hb E heterozygous also showed low MCHC and RBC counts, with other parameters as normal or slightly higher than the normal values. Hb SE is a clinically benign and rare condition with variable clinical manifestations or even asymptomatically [10]. Some may show mild symptoms, and in some cases the condition may be more severe such as hemolytic episodes with frequent blood transfusions, whereas in some cases the person may be asymptomatic other than being anemic, as in the present study. Sudden death of an Hb SE person of 12 years of age after exercise was also reported with ventricular septal cardiac defect at the time of birth, mild asthma, and fractures of radius and ulna 17 months prior to death [15]. These variations in clinical manifestations of the disorder have made its diagnosis difficult. As a result, they are diagnosed occasionally only during examination for other medical complaint [16].

## Conclusion

Hb S is seen among the tribal population of Central India, and Hb E is common among the tribals of Northeastern Region of India. Prevalence of Hb S among the tea tribe population can be attributed to their migration from the central India. This is the first report of occurrence of double heterozygous for Hb S and E among the Proto-Australoid tribal population of this region.

## Data Availability

Data will be made available to the publisher as and when sought.

## Abbreviations

Hb S: Hemoglobin S
Hb E: Hemoglobin E
Hb SE: Hemoglobin SE
Hb C: Hemoglobin C
CBC: Complete blood count
Hb: Hemoglobin
MCV: Mean corpuscular volume
MCH: Mean corpuscular hemoglobin
MCHC: Mean corpuscular hemoglobin concentration
RDW: Red cell distribution width
RBC: Red blood cell
Hb F: Hemoglobin fetal
Hb A_2_: Hemoglobin adult
